# Genetic Configuration of Genomic Resistance Islands in *Acinetobacter baumannii* Clinical Isolates From Egypt

**DOI:** 10.3389/fmicb.2022.878912

**Published:** 2022-07-22

**Authors:** Samira M. Hamed, Amira F. A. Hussein, Mohamed H. Al-Agamy, Hesham H. Radwan, Mai M. Zafer

**Affiliations:** ^1^Department of Microbiology and Immunology, Faculty of Pharmacy, October University for Modern Sciences and Arts (MSA), Giza, Egypt; ^2^Department of Clinical and Chemical Pathology, Faculty of Medicine, Cairo University, Cairo, Egypt; ^3^Department of Pharmaceutics, College of Pharmacy, King Saud University, Riyadh, Saudi Arabia; ^4^Department of Microbiology and Immunology, Faculty of Pharmacy, Al-Azhar University, Cairo, Egypt; ^5^Department of Microbiology and Immunology, Faculty of Pharmacy, Ahram Canadian University, Cairo, Egypt

**Keywords:** *Acinetobacter baumannii*, whole genome sequencing, resistance islands, AbaR4, AbGRI1, AbGRI2, AbGRI3, RI-PER-7

## Abstract

In *Acinetobacter baumannii* (*A. baumannii*), a wide repertoire of resistance genes is often carried within genomic resistance islands (RIs), particularly in high-risk global clones (GCs). As the first in Egypt, the current study aimed at exploring the diversity and genetic configuration of RIs in the clinical isolates of *A. baumannii*. For this purpose, draft genomes of 18 isolates were generated by Illumina sequencing. Disk diffusion susceptibility profiling revealed multidrug resistance (MDR) and extensive drug resistance (XDR) phenotypes in 27.7 and 72.2%, respectively. The highest susceptibility was noted for tigecycline (100.0%) followed by colistin (94.4%), for which an MIC_50_ of 0.25 μg/ml was recorded by the broth microdilution assay. Sequence typing (ST) showed that the majority of the isolates belonged to high-risk global clones (GC1, GC2, and GC9). A novel Oxford sequence type (ST2329) that also formed a novel clonal complex was submitted to the PubMLST database. A novel *bla*_ADC_ variant (*bla*_ADC−258_) was also identified in strain M18 (ST85^Pas^/1089^Oxf^). In addition to a wide array of resistance determinants, whole-genome sequencing (WGS) disclosed at least nine configurations of genomic RIs distributed over 16/18 isolates. GC2 isolates accumulated the largest number of RIs (three RIs/isolate) followed by those that belong to GC1 (two RIs/isolate). In addition to Tn*6022* (44.4%), the *comM* gene was interrupted by AbaR4 (5.5%) and three variants of A. b*aumannii* genomic resistance island 1(AbGRI)-type RIs (44.4%), including AbaR4b (16.6%) and two novel configurations of AbGRI1-like RIs (22.2%). Three of which (AbaR4, AbaR4b, and AbGRI1-like-2) carried *bla*_OXA−23_ within Tn*2006*. With less abundance (38.8%), IS*26*-bound RIs were detected exclusively in GC2 isolates. These included a short version of AbGRI2 (AbGRI2-15) carrying the genes *bla*_TEM−1_ and *aphA1* and two variants of AbGRI3 RIs carrying up to seven resistance genes [*mphE-msrE-armA-sul1-aadA1-catB8-aacA4*]. Confined to GC1 (22.2%), sulfonamide resistance was acquired by an IS*Aba1* bracketed GIsul2 RI. An additional RI (RI-PER-7) was also identified on a plasmid carried by strain M03. Among others, RI-PER-7 carried the resistance genes *armA* and *bla*_PER−7_. Here, we provided a closer view of the diversity and genetic organization of RIs carried by a previously unexplored population of *A. baumannii*.

## Introduction

In the last decades, *Acinetobacter baumannii* infections have moved to the forefront of challenges encountered by clinicians worldwide. It is mainly recognized for causing a wide range of difficult-to-treat hospital-acquired infections, particularly in critically ill patients (Morris et al., 2019). Working in concert, the remarkable capacity for upregulating intrinsic resistance mechanisms and acquisition of foreign resistance genes contributed to an ever-expanding spectrum of antimicrobial resistance in *A. baumannii*. Leaving behind limited or no antimicrobial treatment options, extensively drug-resistant (XDR) and pandrug-resistant strains have been increasingly reported from different parts of the world (Hsueh et al., 2002; Leite et al., 2016; Hamidian and Nigro, 2019). Most of them are members of the high-risk global clones (GCs) 1 and 2 (also known as international clones; ICs) (Karah et al., 2012). Genome sequencing of the earliest strains of the high-risk GCs uncovered a wide repertoire of resistance genes being associated with genomic resistance islands (RIs) (Hamidian and Hall, 2017b). These are genomic regions encompassing variable assortments of transposons and integrons loaded with specific resistance genes (Fournier et al., 2006). They are one of the hallmarks of the horizontal transfer of resistance genes (Carraro et al., 2017).

The first known genomic RI, designated AbaR1 (A. ba*umannii* Resistance 1), was identified in *A. baumannii* strain AYE from France carrying antimicrobial and heavy metal resistance genes within transposon fragments (Fournier et al., 2006). With a wide variability in size, genetic structure, and insertion sites (Bi et al., 2020), at least seven families of genomic RIs are currently known. These include AbaR-type islands (Post et al., 2010), AbaR4 (Hamidian and Hall, 2011), and A. b*aumannii* genomic resistance islands (AbGRIs) types 1 to 5 (Nigro and Hall, 2012b; Nigro et al., 2013; Wright et al., 2014; Blackwell et al., 2017; Chan et al., 2020; Hua et al., 2021). Any or more than one RIs may be carried by MDR *A. baumannii* strains (Chan et al., 2015; Hamidian and Hall, 2017b). In addition to the Tn*6019* backbone, AbaR contains multiple antibiotic resistance regions (MARRs) enclosed by two copies of Tn*6018*. AbaR-type RIs are commonly inserted within the ATPase-coding gene *comM* (Hamidian and Hall, 2018) often in GC1 strains. In the same location, two other RIs were identified. These include AbaR4, in which Tn*2006* is inserted in a Tn*6022* backbone (Hamidian and Hall, 2011), and AbGRI1, identified in GC2 strains (Nigro and Hall, 2012a,b; Hamidian and Hall, 2017b). AbGRI1 consists of Tn*6022* (or its deletion derivatives) and Tn*6172* joined by a plasmid-derived linker (Hamidian and Hall, 2017b). AbGRI1 variants often carry the resistance genes *sul2, tet(B), strA, strB*, and sometimes *bla*_OXA−23_ (Bi et al., 2020). The three RIs, AbaR, AbaR4, and AbGRI1, are complex class III transposons carrying the transposition genes *tniCABDE* that target the *comM* gene for insertion (Hamidian and Hall, 2017b). The other four types of AbGRIs are IS*26*-bound transposons harboring variable combinations of resistance genes that are characteristic for each type. They are commonly identified in the chromosomes of *A. baumannii* strains that belong to GC2. They include AbGRI2 (Nigro et al., 2013), AbGRI3 (Blackwell et al., 2017), AbGRI4 (Chan et al., 2020), and AbGRI5 (Hua et al., 2021). AbGRI2 characteristically carries all or some of the resistance genes *bla*_TEM_, *aphA1, catA1*, and a class I integron carrying *sul1, aacC1*, and *aadA1* (Nigro et al., 2013). AbGRI3 commonly inserts within a putative GNAT family N-acetyltransferase gene. In addition to *armA* conferring resistance to all clinically useful aminoglycosides, AbGRI3 also carries *msrE* and *mphE*, with or without class I integron carrying the resistance genes *aacA4, catB8, aadA*, and *sul1*. In some cases, IS*26*-bracketed *aphA1* also integrates into AbGRI3 (Blackwell et al., 2017). Recently, AbGRI4 was identified in GC2 and non-GC2 strains carrying the resistance genes *aadB, aadA2*, and *sul1* in a class I integron. AbGRI4 uniquely targets an α/β-hydrolase gene (Chan et al., 2020). AbGRI5 is the latest RI to be identified in *A. baumanni* that resembles AbGRI3 in harboring *armA, msrE*-*mphE, sul1*, and class I integron that carries a different array of resistance genes [*bla*_PER−1_-*bla*_CARB−2_-*aadA2*-*cmlA1*-*aadA1*] compared to AbGRI3. In addition, AbGRI5 distinctively carries the macrolide resistance gene *ere(B)* (Hua et al., 2021).

Even though reports about the structure of RIs carried by strains of this extremely problematic pathogen were published from several parts of the world (Lee et al., 2016; Blackwell et al., 2017; Kim et al., 2017; Chan et al., 2020; Leal et al., 2020; Hua et al., 2021), little is known about those circulating in Egyptian hospitals. Here, we used whole-genome sequencing (WGS) to analyze the diversity and configuration of RIs carried by 18 strains of *A. baumannii* isolated from patients admitted to one of the largest tertiary university hospitals in Cairo, Egypt, in 2020.

## Materials and Methods

### Clinical Isolates

The current study included 20 non-duplicate clinical isolates of carbapenem-resistant *A. baumannii* from patients admitted to Kasr Al-Ainy University Hospital, Cairo, Egypt. The isolates were recovered from clinical specimens received by the clinical pathology laboratory for bacteriological analysis in the period from July to October 2020. They were identified to species level using the VITEK^®^2 automated identification system (bioMérieux, Marcy l'Etoile, France) before polymerase chain reaction (PCR) amplification of the *bla*_OXA−51-like_ genes, as described before (Turton et al., 2006). Identification was further confirmed by WGS using the Speciesfinder tool hosted by the Center of Genomic Epidemiology (http://www.genomicepidemiology.org/).

### Antimicrobial Susceptibility Testing

Broth microdilution assay was used for the determination of the minimum inhibitory concentrations (MICs) of colistin (Sigma-Aldrich, St Louis, MO, USA) in a concentration range of 128-0.125 μg/ml. Susceptibility to other antimicrobial agents was inferred by Kirby–Bauer disc diffusion test. These included amikacin (30 μg), amoxicillin/clavulanic acid (20/10 μg), ampicillin (10 μg), cefepime (30 μg), cefotaxime (30 μg), cefoxitin (30 μg), ceftriaxone (30 μg), imipenem (10 μg), levofloxacin (5 μg), meropenem (10 μg), piperacillin/tazobactam (10/100 μg), tetracycline (30 μg), tigecycline (15 μg), and trimethoprim/sulfamethoxazole (1.25/23.75 μg). Both susceptibility tests were performed and interpreted according to the Clinical and Laboratory Standards Institute (CLSI) guidelines (CLSI, 2020) for all antimicrobial agents except tigecycline for which susceptibility breakpoints recommended by EUCAST v11.0 for *Enterobacterales* were used (EUCAST, 2021). For quality control purposes, *Escherichia coli* ATCC 25922 and *Pseudomonas aeruginosa* ATCC 27853 were used.

### Whole-Genome Sequencing

After DNA extraction using the QIAGEN DNA purification kit (Qiagen, Valencia, CA) and library preparation using the Nextera DNA Sample Preparation kit (Nextera, USA), WGS was performed on an Illumina MiSeq platform (Illumina Inc., San Diego, CA, USA). Pre-assembly processing of the generated reads was carried out by FastQC (Andrews, 2010) for quality assessment and Trimmomatic v0.32 (Bolger et al., 2014) for the trimming of low-quality reads. *De novo* assembly of trimmed reads was carried out using SPAdes 3.14.1 (Bankevich et al., 2012). Post-assembly metrics were generated by QUAST v5.0.2 (Gurevich et al., 2013). Draft genomes were annotated using the NCBI Prokaryotic Genome Annotation Pipeline (PGAP) (Tatusova et al., 2016). Plasmids were assembled from Illumina reads using PlasmidSPAdes (Antipov et al., 2016), a software for reading coverage-assisted plasmid identification. Assembly graphs (Fastg files) generated by PlasmidSPAdes were visualized on a bandage (Wick et al., 2015). Plasmid sequences were extracted from circular contigs or groups of contigs forming circular paths containing plasmid replication and/or mobilization genes. Contigs forming circular but overlapping paths were BLASTed for closest plasmids that were subsequently used for reference mapping using the short reads mapping tool BWA-MEM (Li and Durbin, 2010).

### Epidemiology Analysis

Two sequence-based typing methods were used for the epidemiology analysis of the isolates. These included multilocus sequence typing (MLST) and single-nucleotide polymorphism (SNP)-based phylogeny analysis.

The draft genomes were uploaded to the PubMLST server (https://pubmlst.org/abaumannii/) for assigning STs for the isolates according to both Pasteur (Diancourt et al., 2010) and Oxford schemes (Bartual et al., 2005). Allocation of the isolates into clonal complexes (CCs) was done by goeBURST analysis. For this purpose, all allelic profiles defined by both schemes were retrieved from the PubMLST database (accessed on 30 April 2021). Together with the allelic profiles of the isolates studied here, they were used as inputs for Phyloviz software for the generation of minimum spanning trees using the goeBURST algorithm (Ribeiro-Goncalves et al., 2016).

Using the default setting parameters, the CSI phylogeny 1.4 online tool (https://cge.cbs.dtu.dk/services/CSIPhylogeny/) was employed in inferring the phylogeny of the isolates based on the concatenated alignment of the high-quality SNPs. *A. baumannii* ATCC17978 was used as a reference for the analysis. The analysis initially included 44 complete and draft genomes of *A. baumannii* strains that belong to STs identified here obtained from the NCBI and PubMLST databases. For easier visualization, the final phylogenetic tree was constructed using a smaller number of genomes of *A. baumannii* strains that were clustered with our isolates. Interactive tree of life (iTOL) v3 software (https://itol.embl.de/) (Letunic and Bork, 2016) was used for visualization and editing of the phylogenetic tree.

### Antimicrobial Resistance Determinants and Resistance Island Analyses

Genes with a minimum of 80% coverage and 95% identity to known resistance genes were identified using the Comprehensive Antibiotic Resistance Database (CARD) server (https://card.mcmaster.ca/analyze/rgi) (Alcock et al., 2020). Point mutations of the genes relevant to fluoroquinolones (*gyrA* and *parC*) and colistin (*lpxACD* and *pmrABC*) resistance were extracted from the assemblies for pairwise comparison to the corresponding genes of the reference strain *A. baumannii* ATCC 19606 (GenBank accession: CP045110.1).

The context of resistance genes was examined by visualizing the annotated contigs using SnapGene software v5.1.3.1 by Insightful Science (http://www.snapgene.com). Insertion sequences (ISs) were identified by BLAST analysis against the nucleotide database of the NCBI and novel ISs were named by the ISFinder database team (http://www-is.biotoul.fr). Resistance islands were predicted using the webserver IslandViewer4 webtool (http://www.pathogenomics.sfu.ca/islandviewer/) (Bertelli et al., 2017), through which the draft genomes were mapped against different reference genomes. For RIs fragmented into multiple contigs, assembly gaps were filled by mapping raw reads against the closest RI using BWA (Li and Durbin, 2010).

### Accession Numbers

The Whole Genome Shotgun project including Fastq files generated by the Illumina sequencer and the assembled draft genomes were submitted to GenBank database under the BioProject number PRJNA690827. The nucleotide sequence of the novel *bla*_ADC−258_ variant was submitted to the NCBI GenBank database under the accession number (MZ224612.1).

## Results

### Bacterial Strains and Clinical Data

Twenty carbapenem-resistant *A. baumannii* isolates were received by the clinical pathology laboratory of Kasr Al-Ainy University Hospital, Cairo, Egypt, during the study period. All were preserved with the purpose of a WGS-based analysis of RIs. Having successfully passed the post-assembly quality control criteria, only 18 strains were selected for further analysis. Post-assembly and annotation metrics of the generated draft genomes are shown in Supplementary Table 1. Half of the strains selected for the study were isolated from patients in critical care units and at least 22.2% were from pediatric patients. Clinical data of all isolates are shown in Table 1.

**Table 1:** Demographic data.

**Strain**	**Specimen**	**Age**	**Gender**	**Diagnosis**	**Hospital Unit**
M01	Endotracheal tube	5 Ds	Male	Chest infection	NICU
M02	Wound swab	28 Ys	Female	Sub ovarian abscess removal	ICU
M03	Blood	48 Ys	Male	Fever	Internal Medicine
M04	Sputum	NA	Female	Chest infection	ER
M05	Blood	NA	Female	Ventilator-associated pneumonia	Chest ICU
M06	Sputum	NA	Male	Pneumonia	ER
M09	Blood	NA	Female	Fever	ER
M10	Blood	24 Ds	Female	Pneumonia	NICU
M11	Pleural fluid	20 Ds	Female	Pneumonia	NICU
M12	Blood	50 Ys	Female	Fever of unknown origin	ER
M13	Wound swab	34 Ys	Male	Fever	ICU
M14	Urine	60 Ys	Male	Fever	ER
M15	Wound swab	NA	Male	Burn	Burns
M16	Blood	NA	Male	Fever	ER
M17	Sputum	56 Ys	Female	Pneumonia	ICU
M18	Blood	55 Ys	Female	Fever and disturbed consciousness level	ER
M19	Blood	20 Ds	Female	Fever of unknown origin	ICU
M20	Blood	65 Ys	Male	Splenectomy and fever	ICU

*Ds, days; Ys, years; NA, not available; NICU, neonatal intensive care unit; ICU, intensive care unit; ER, emergency department*.

### Molecular Epidemiology

**Figure 1:**
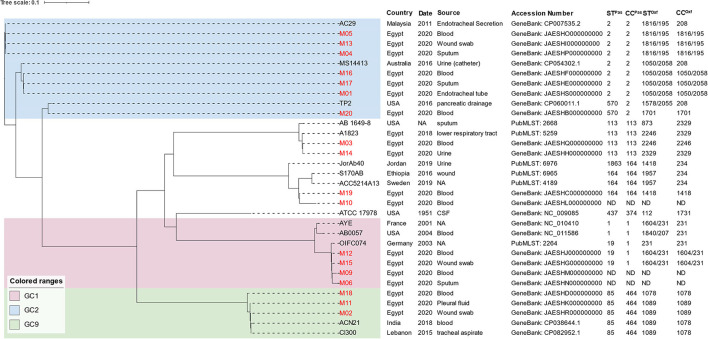
SNP-based phylogenetic tree of *A. baumannii* draft genomes sequenced in the current study compared to other strains obtained from the NCBI and PubMLST databases. Labels of the *A. baumannii* strains sequenced in the current study are written in red. ST^Pas^, sequence type according to Pasteur scheme; ST^Oxf^, sequence type according to Oxford scheme; GC, global clone.

The MLST analysis revealed that the isolates belonged to six Pasteur and nine Oxford STs (Figure 1). GoeBURST analysis (Supplementary Figure 1) showed that the majority of the isolates belonged to the high-risk global clones 1, 2, and 9. Predominantly, seven isolates (38.8%) belonged to GC2 distributed over two CCs (CC208 and CC546) according to the Oxford scheme. GC9 (CC464^Pas^/1078^Oxf^) and GC1 (CC1^Pas^/231^Oxf^) were represented by three and two isolates, respectively. M14 had a novel Oxford ST (2329) that also formed a novel clonal complex to which 11 STs including that of M03 (ST2246^Oxf^) belonged. The SNP-based phylogenetic analysis generated a seven-cluster phylogenetic tree (Figure 1). Notably, isolates that shared an Oxford ST were clustered together. The isolates M06 and M09 with undetermined STs were found to be phylogenetically related to GC1 isolates. The tree also showed that our isolates were clustered with other strains isolated in different parts of the world.

### Antimicrobial Susceptibility Profiles and Resistance Determinants

**Figure 2:**
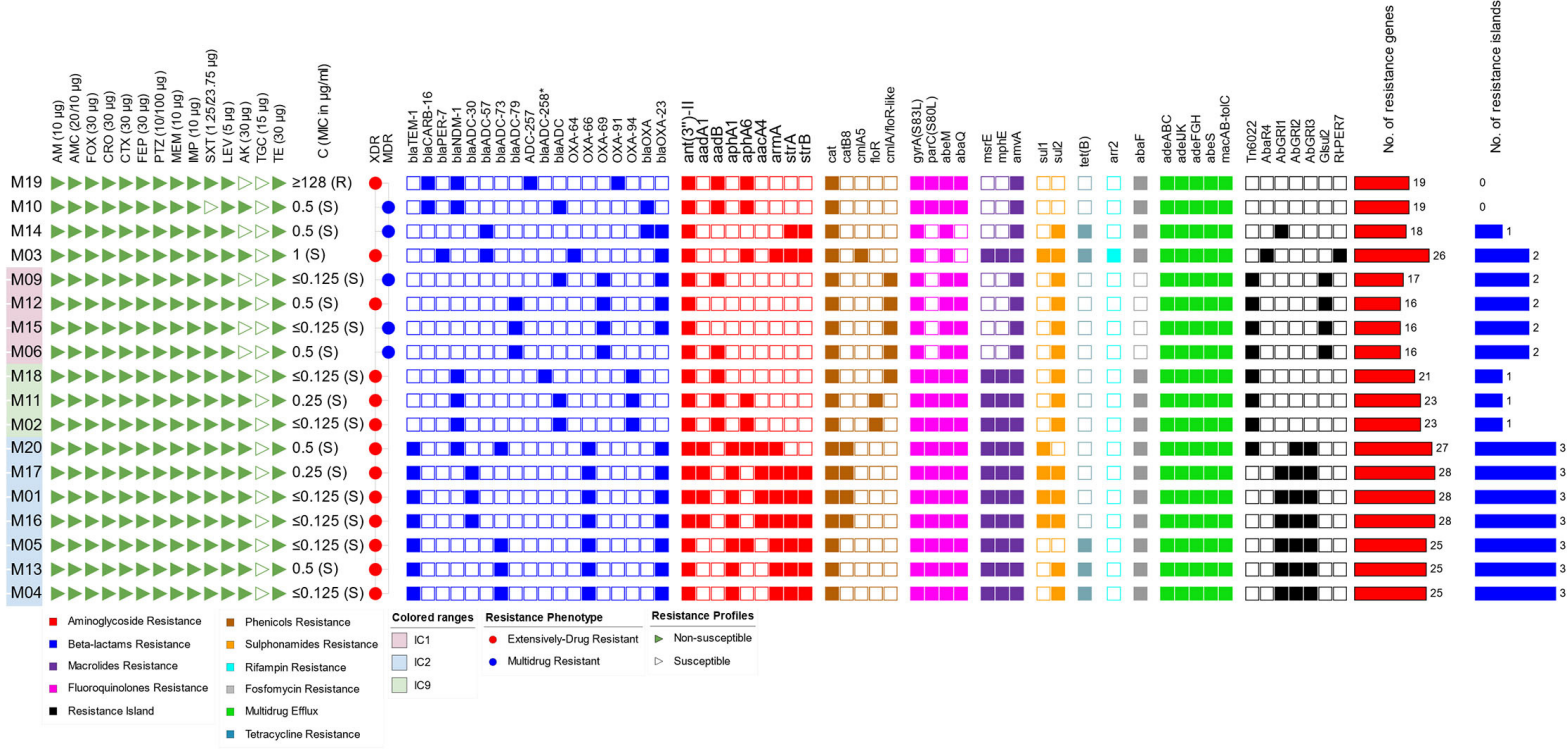
Overview of the antimicrobial susceptibility profiles and resistance determinants. The isolates were ordered according to their SNP-based phylogenetic relationship. Labels of GC1 isolates (together with their phylogenetically related isolates) are highlighted in pink and those of GC2 and GC9 are highlighted in blue and green, respectively. The map is divided into panels corresponding to susceptibility to antimicrobial agents (triangular icons), resistance phenotypes (circular icons), resistance determinants (square icons), and the number of resistance determinants and islands (bars). AM, ampicillin; AMC, amoxicillin/clavulanic acid; FOX, cefoxitin; CRO, ceftriaxone; CTX, cefotaxime; FEP, cefepime; PTZ, piperacillin/tazobactam; MEM, meropenem; IMP, imipenem; SXT, sulfamethoxazole/trimethoprim; LEV, levofloxacin; AK, amikacin; TGC, tigecycline; TE, tetracycline; C, colistin; XDR, extensive drug resistance; MDR, multidrug resistance.

Based on the definitions proposed by Magiorakos et al. (2012) for MDR and XDR, the majority of the isolates were XDR (13/18, 72.2%), and only five isolates (27.7%) showed an MDR phenotype. All GC2 and GC9 isolates were XDR, while MDR isolates belonged to GC1 as well as STs that do not belonging to high-risk clones. Susceptibility to tigecycline was retained by all isolates. Except for one isolate (5.5%), all were susceptible to colistin with an MIC_50_ of 0.25 μg/ml. Only five isolates (27.7%) were susceptible to amikacin, and one isolate (5.5%) was susceptible to trimethoprim/sulfamethoxazole. All isolates were nonsusceptible to all other tested antimicrobials. A wide repertoire of resistance genes was identified in our isolates, most of which were associated with mobile elements. As many as 38 resistance determinants were identified in combinations of up to 28 determinants per isolate. These included genes coding for antibiotic inactivation, target protection, target alteration, target replacement, and antibiotic efflux. The largest number of co-existing resistance genes was found in GC2 isolates (25-28 determinants/isolate), followed by those that belonged to GC9 (21–23 determinants/isolate). Antimicrobial susceptibility profiles and resistance determinants of all isolates are shown in Figure 2.

#### Resistance to β-Lactams

In addition to the intrinsic resistance genes (*bla*_ADC_ and *bla*_OXA−51−like_) to β-lactams, five acquired β-lactamase-coding genes, encompassing *bla*_TEM−1_, *bla*_CARB−16_, *bla*_NDM−1_, *bla*_PER−7_, and *bla*_OXA−23_, were identified. Up to five β-lactamase coding genes co-existed in the tested isolates. At least six *bla*_ADC_ variants were identified, including a novel variant (*bla*_ADC−258_) carried by M18. *bla*_ADC−258_ showed 99.74% similarity to *bla*_ADC−176_ with the amino acid alterations Q2R and D24G. Meanwhile, *bla*_ADC_ variants carried by four isolates could not be identified due to insertion sequence (IS) interruption (M02 and M11) or assembly gaps (M09 and M10). The N terminus of the *bla*_ADC−73_ carried by M20 was interrupted by an unknown sequence, as described before (Zafer et al., 2021). Interestingly, isolates of the same Oxford ST carried the same *bla*_ADC_ variants. An upstream IS*Aba1* was confirmed for only eight isolates (M01, M04, M06, M12, M13, M15, M16, and M17), all belonging to GCs 1 and 2. On their chromosomes, the isolates also carried five alleles of the intrinsic β-lactamase-coding gene *bla*_OXA−51−like_. Isolates of the same clonal complex (Pasteur or Oxford) shared the same *bla*_OXA−51−like_ variant. Of all acquired β-lactamase-coding genes, *bla*_OXA−23_ (class D β-lactamase-coding gene) was the most prevalent (12/18, 66.6%) either within RIs (5/18, 27.7%) or more frequently bracketed by IS*Aba1* in Tn*2006* (7/18, 38.8%). The *bla*_OXA−23_-positive isolates belonged to GC2 and GC1, and two isolates (M03 and M14) belonged to the novel CC113^Pas^/2329${}_{.}^{\text{Oxf}}$ The gene *bla*_OXA−23_ was carried within Tn*2006* in GC1 isolates and GC2 isolates that belonged to the Oxford STs ST1050/2058 and ST1701. Meanwhile, in GC2 isolates of the ST1816/195^Oxf^, *bla*_OXA−23_ was hosted by AbaR4b. M03 and M14 carried *bla*_OXA−23_ within AbaR4 and an AbGRI1-like-2 RI, respectively. Harbored by an AbGRI2-15 and exclusively in GC2, the class A β-lactamase-coding gene *bla*_TEM−1_ was found in seven isolates (38.8%). Among our isolates were six (33.3%) NDM-1 producers. These included all GC9 isolates, one GC2 isolate (M20), as well as M19 (ST164^Pas^/1418^Oxf^) and its phylogenetically related isolate M10. The genetic environment of *bla*_NDM−1_ was described in our previous study (Zafer et al., 2021). We reported, for the first time, a novel transposon in which both *bla*_NDM−1_ and *aphA6* were enclosed by two direct copies of IS*Aba14*. The transposition potential of the transposon was later demonstrated using bioinformatic tools (unpublished data). This environment was described only for GC9 isolates that belonged to ST1089^Oxf^ as well as M10. While the right arm of the IS*Aba14*-bracketed transposon carrying *bla*_NDM−1_ was found in other NDM producers, the full sequence of the transposon could not be spotted.

The isolates M19 and M10 also carried *bla*_CARB−16_ in contigs showing 100% similarity to a 63,650 kb plasmid carried by *A. baumannii* strain DT01139C (GenBank accession: CP053220.1) isolated in Tanzania in 2017. However, *bla*_CARB−16_-positive plasmids could not be identified either by PlasmidSPAdes *de novo* assembly or by mapping the raw reads against the DT01139C plasmid. Finally, with the lowest prevalence, *bla*_PER−7_ was identified only in M03. Together with six more resistance genes, *bla*_PER−7_ was carried within RI-PER-7.

#### Resistance to Aminoglycosides

**Figure 3:**
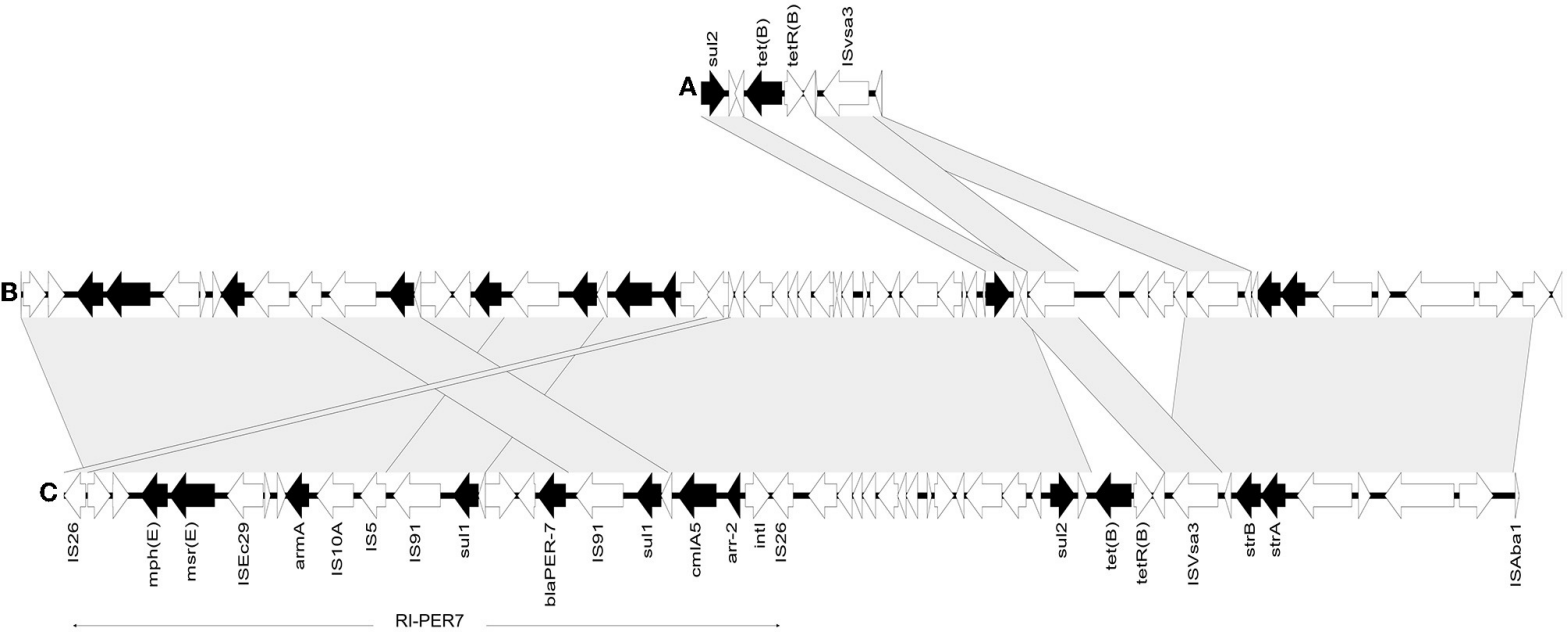
Comparative genomic analysis of the partial sequence of M03 plasmid and the closely similar plasmid pPM194229_1(CP050433.1). **(A)** pPM194229_1 plasmid sequence region [218446-224334 bp]. **(B)** pPM194229_1 plasmid sequence region [1-50187 bp]. **(C)** M03 plasmid partial sequence. Arrows correspond to open reading frames, and black ones denote resistance genes. Gray shadings highlight regions of 99% or more nucleotide identity.

Resistance genes to aminoglycosides were found in abundance in our collection. Of them, *armA*, conferring resistance to all clinically relevant aminoglycosides, was the most prevalent and was carried by 8/18 (44.4%) isolates. These included all GC2 isolates in which it was carried on AbGRI3 and M03 in which *armA* was hosted by RI-PER-7. The amikacin resistance gene *aphA6* was carried by 7/18 (38.8%) isolates that belonged to different STs. Most commonly, *aphA6* was bracketed by IS*Aba14* and IS*Aba125* in the *bla*_NDM−1_-positive isolates either within the composite IS*Aba14* bracketed transposon described above (M02, M10, and M11) or not (M19 and M20). In M05, *aphA6* was carried on a 70,101-bp RepAci6 plasmid closely similar to pACICU2 (GenBank accession: CP031382.1). The context of *aphA6* in M03 could not be defined. On pRAY plasmid derivatives, *aadB* was carried by 7/18 (38.8%) isolates that belonged to different sequence types except for those of GC2. Within AbGRI3, GC2 isolates of the STs ST1050/2058^Oxf^ and ST1701^Oxf^ carried *aacA4* and *aadA1* that were undetectable in other GC2 strains carrying a shorter version of AbGRI3. Other detected aminoglycoside resistance genes included the intrinsic gene *ant(3”)-II* (Zhang et al., 2017) and other acquired resistance genes, such as *aphA1*(38.8%%)*, strA* (44.4%), *strB* (44.4%), and *aadA1* (22.2%). In all *aphA1*-positive isolates that all belonged to GC2, the gene was carried on AbGRI2-15 together with *bla*_TEM−1_. The genes *strA* and *strB* co-existed in all GC2 isolates that belonged to CC208^Oxf^ in which they were carried on variants of AbGRI1. They also co-existed in CC113^Pas^/2329^Oxf^ isolates (M03 and M14) but within different environments. In M14, *strA* and *strB* were carried on AbGRI1-type RI. In M03, they were harbored by a transposon (~ 24 Kb) also carrying *sul2* and *tet(B)* and bracketed by IS*Aba1* and IS*26* that were shared with an adjacent RI-PER-7. The whole genetic structure showed 99.9% similarity to a 226,394-bp plasmid pPM194229_1 (GenBank accession: CP050433.1). Using the plasmid sequence as a reference for mapping produced a sequence with 99.99% identity to the reference plasmid with a coverage of 90.0%. Although the full sequence of the plasmid could not be identified, the *de novo* assembled fragment revealed genetic rearrangement compared to the closely matched plasmid. This included insertion of [*tet(B)*-*tetR(B)*] downstream to *sul2* through homologous recombination that was also associated with IS*Vsa3*-mediated deletion, as shown in Figure 3. The context of β-lactams and aminoglycoside resistance determinants in all isolates are summarized in Table 2.

**Table 2:** β-lactam and aminoglycoside resistance determinants and their context.

**Isolate No**.	**Resistance to** **β-lactams**		**Resistance to aminoglycosides**	
	**Intrinsic resistance genes (An upstream IS*Aba1*)**	**Acquired resistance genes (associated MGEs)**	**Intrinsic resistance genes**	**Acquired resistance genes (associated MGEs)**
M01	*bla*_ADC−30_ (present) *bla*_OXA−66_ (ND)	*bla*_OXA−23_ (Tn*2006*) *bla*_TEM−1_ (AbGRI2-15)	*ant(3”)-II*	*aacA4* (AbGRI3-1) *aadA1* (AbGRI3-1) *aphA1* (AbGRI2-15) *armA* (AbGRI3-1) *strA* (AbGRI1-like-1) *strB* (AbGRI1-like-1)
M02	*bla*_ADC_ (interrupted by IS*1008*) *bla*_OXA−94_ (present)	*bla*_NDM−1_ (IS*Aba14* bracketed transposon also carrying *aphA6*)		*aadB* (pRAY*, JQ904627.1) *aphA6* (IS*Aba14* bracketed transposon also carrying *bla*_NDM−1_)
M03	*bla*_ADC−57_ (ND) *bla*_OXA−64_ (ND)	*bla*_OXA−23_ (AbaR4) *bla*_PER−7_ (RI-PER-7, in an undetermined plasmid closely similar to pPM194229_1)		*aphA6* (ND) *armA* (RI-PER-7, in a plasmid closely similar to pPM194229_1) *strA* (plasmid closely similar to pPM194229_1) *strB* (plasmid closely similar to pPM194229_1)
M04	*bla*_ADC−73_ (present) *bla*_OXA−66_ (absent)	*bla*_OXA−23_ (AbaR4b) *bla*_TEM−1_ (AbGRI2-15)		*aphA1* (AbGRI2-15) *armA* (AbGRI3-1) *strA* (AbaR4b) *strB* (AbaR4b)
M05	*bla*_ADC−73_ (ND) *bla*_OXA−66_ (absent)	*bla*_OXA−23_ (AbaR4b) *bla*_TEM−1_ (AbGRI2-15)		*aphA1* (AbGRI2-15) *aphA6* (RepAci6 plasmid 99.9% similar to pACICU2) *armA* (AbGRI3-1) *strA* (AbaR4b) *strB* (AbaR4b)
M06	*bla*_ADC−79_ (present) *bla*_OXA−69_ (ND)	None		*aadB* (pRAY*-like,99.98% similarity)
M09	*bla*_ADC_ (ND) *bla*_OXA−69_ (ND)	*bla*_OXA−23_ (Tn*2006*)		*aadB* (pRAY*, JQ904627.1)
M10	*bla*_ADC_ (ND) *bla*_OXA_ (ND)	*bla*_NDM−1_ (IS*Aba14* bracketed transposon also carrying *aphA6*) *bla*_CARB−16_ (ND)		*aadB* (pRAY*-V1, JF343536.2) *aphA6* (IS*Aba14* bracketed transposon also carrying *bla*_NDM−1_)
M11	*bla*_ADC_ (interrupted by IS*1008*) *bla*_OXA−94_ (present)	*bla*_NDM−1_ (IS*Aba14* bracketed transposon also carrying *aphA6*)		*aadB* (pRAY*, JQ904627.1) *aphA6* (IS*Aba14* bracketed transposon also carrying *bla*_NDM−1_)
M12	*bla*_ADC−79_ (present)	*bla*_OXA−23_ (Tn*2006*)		None
	*bla*_OXA−69_ (absent)			
M13	*bla*_ADC−73_ (present)	*bla*_OXA−23_ (AbaR4b) *bla*_TEM−1_ (AbGRI2-15)		*aphA1* (AbGRI2-15) *armA* (AbGRI3-1) *strA* (AbaR4b) *strB* (AbaR4b)
	*bla*_OXA−66_ (ND)			
M14	*bla*_ADC−57_ (ND) *bla*_OXA−51−like_ (ND)	*bla*_OXA−23_ (AbGRI1-like-2)		*strA* (AbGRI1-like-2) *strB* (AbGRI1-like-2)
M15	*bla*_ADC−79_ (present) *bla*_OXA−69_ (absent)	*bla*_OXA−23_ (Tn*2006*)		None
M16	*bla*_ADC−30_ (present) *bla*_OXA−66_ (absent)	*bla*_OXA−23_ (Tn*2006*) *bla*_TEM−1_ (AbGRI2-15)		*aacA4* (AbGRI3-1) *aadA1* (AbGRI3-1) *aphA1* (AbGRI2-15) *armA* (AbGRI3-1) *strA* (AbGRI1-like-1) *strB* (AbGRI1-like-1)
M17	*bla*_ADC−30_ (present) *bla*_OXA−66_ (absent)	*bla*_OXA−23_ (Tn*2006*) *bla*_TEM−1_ (AbGRI2-15)		*aacA4* (AbGRI3-1) *aadA1* (AbGRI3-1) *aphA1* (AbGRI2-15)
				*armA* (AbGRI3-1) *strA* (AbGRI1-like-1) *strB* (AbGRI1-like-1)
M18	*bla*_ADC−258_*^*a*^* (absent) *bla*_OXA−94_ (absent)	*bla*_NDM−1_ (IS*Aba14* interrupted Tn*125*)		*aadB* (pRAY*, JQ904627.1)
M19	*bla*_ADC−257_ (absent) *bla*_OXA−91_ (absent)	*bla*_NDM−1_ (IS*Aba14* interrupted Tn*125*) *bla*_CARB−16_ (ND)		*aadB* (pRAY*-V1, JF343536.2) *aphA6* (IS*Aba14*-*aphA6*-IS*Aba125*)
M20	*bla*_ADC−73_ (absent) *bla*_OXA−66_ (absent)	*bla*_NDM−1_ (IS*Aba14* interrupted Tn*125*) *bla*_TEM−1_ (AbGRI2-15) *bla*_OXA−23_ (Tn*2006*)		*aphA1* (AbGRI2-15) *aphA6* (IS*Aba14*-*aphA6*-IS*Aba125*) *aadA1*(AbGRI3-4) *aacA4* (AbGRI3-4) *armA* (AbGRI3-4)

^a^novel variant; ND, could not be determined; MGEs, mobile genetic elements. The symbol * is part of the plasmid's name.

Isolates carrying single mutations (*gyrA*; S83L) belonged to ST113^Oxf^ (M03 and M14) and ST231^Oxf^ (M12 and M15) and their phylogenetically related strains (M06 and M09). Genes coding the quinolone efflux pumps *abeM* and *abaQ* were found in 100.0% and 88.8% of the isolates, respectively.

The chloramphenicol acetyltransferase-coding gene *cat* was carried in the chromosomes of all isolates not associated with any mobile elements. Meanwhile, *catB8* was identified in GC2 isolates within AbGRI3 except those carrying the short version of the island (M04, M05, and M13). Genes coding the chloramphenicol efflux pumps FloR and CmlA5 were also identified. FloR efflux pump-coding gene was carried by M02 and M11 (2/18, 11.1%) in association with *sul2* gene and ISs, as described before (Zafer et al., 2021). A novel *cmlA/floR*-like gene variant was identified in five isolates (27.7%). These included GC1 isolates M06, M09, M12, and M15 and the GC9 isolate M18. The gene was associated with a novel downstream 1,206-bp long IS designated IS*Aba61*. Together with the passenger *cmlA/floR*-like gene, IS*Aba61* was distinctively inserted within a molybdopterin-dependent oxidoreductase-coding gene, as shown in Supplementary Figure 2. As a transposition signature, the insertion of IS*Aba61* generated an 8-bp TGAAAATA duplication in the target site. Only one isolate (M03) carried *cmlA5* within RI-PER-7.

In addition to the efflux pump gene *amvA*, macrolide resistance was coded by *msrE* and *mphE* that co-existed in 61.1% of the isolates. They were associated with the resistance islands AbGRI3 and RI-PER-7 in GC2 isolates and M03, respectively. In GC9 isolates, *msrE* and *mphE* were located outside the RIs enclosed by the insertion sequences ISNCY and IS*Aba1* in the upstream and downstream regions, respectively.

As many as 15 isolates (83.3%) carried at least one sulfonamide resistance gene. More frequently, the isolates carried *sul2*, which was identified in 14 isolates with no clonal bias. The gene was hosted by two types of RIs, namely, GIsul2 in GC1 (M12 and M15) and phylogenetically related isolates (M06 and M09) and AbGRI1-type RIs in M01, M14, M16, and M17. Only five isolates carried *sul1* that was associated with AbGRI3 in GC2 isolates (Oxford STs ST1050/2058 and ST1701) and RI-PER-7 in M03.

#### Resistance to Other Antimicrobial Classes

Resistance to levofloxacin was associated with *gyrA* mutations encoding S83L amino acid alterations in all isolates. Meanwhile, missense mutations (S80L) in the topoisomerase-coding gene (*parC*) were identified in only 12 (66.6%) isolates.

The gene *tet(B)* was the only tetracycline resistance gene identified in the isolates (5/18, 27.7%). In association with the resistance genes *strA, strB*, and *bla*_OXA−23_, it was located within AbGRI1 in all GC1 isolates and M14. A different environment was found for *tet(B)* in M03 in which it was carried on a plasmid whose sequence was partially identified, as described above. Except for GC1 isolates, genes coding the fosfomycin major facilitator superfamily (MFS) transporter AbaF were carried on the chromosomes of all isolates not associated with any mobile elements. Within RI-PER-7, the rifampin resistance gene *arr-2* was only carried by M03. Colistin resistance in M19 was found to be associated with mutations in *pmrB* (H89L), *pmrC* (I42V, I212V, R323K, A354S, and V470I), *IpxA* (Y131H and Y231H), *IpxC* (C120R, N287D, and K130T), and *lpxD* (V631 and E117K), as described before (Zafer et al., 2021).

### Resistance Islands (RIs)

Whole-genome sequencing of the isolates disclosed at least nine configurations of genomic RIs. An additional RI, RI-PER-7, was found to be carried on a plasmid whose structure was only partially identified. GC2 isolates accumulated the largest number of RIs (three RIs/isolate) followed by GC1 isolates in which two genomic RIs co-existed.

**Figure 4:**
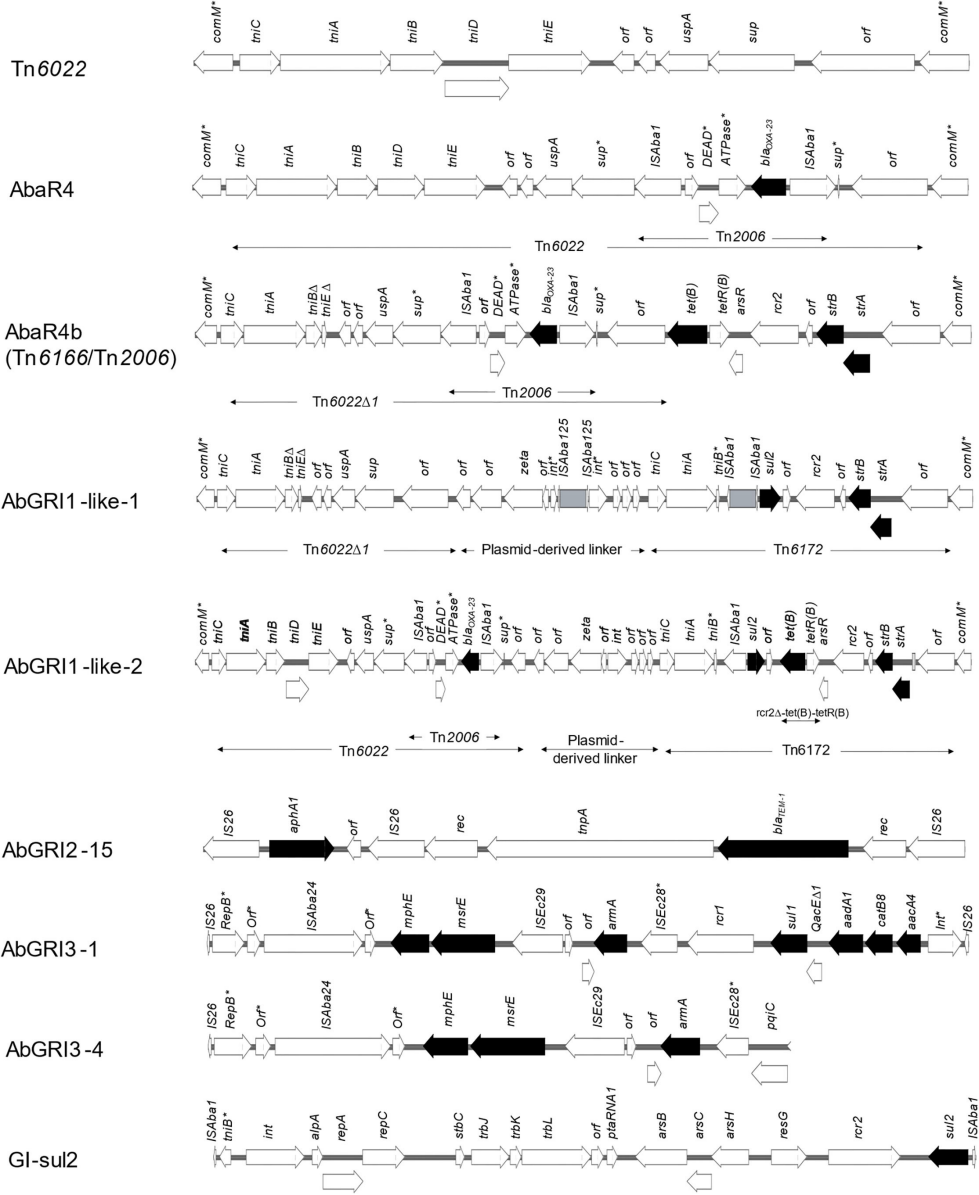
Structures of resistance islands identified in the current study. Nine types of genomic RIs were identified in the isolates. These included Tn*6022*, AbaR4, three AbGRI1 variants, one AbGRI2, two variants of AbGRI3, and GIsul2. Arrows denote open reading frames. Antimicrobial resistance genes are represented by black arrows. Asterisks indicate interrupted genes.

For the detection of AbaR-, AbaR4-, and AbGRI-type RIs, *comM* gene integrity was checked in all isolates. The gene was found to be interrupted in 16/18 (88.8%) isolates (all except M10 and M19) most frequently by Tn*6022* (the backbone of AbaR4). Lacking any resistance genes, Tn*6022* was carried by eight isolates (44.4%), including all GC1 and GC9 isolates as well as M20 that belonged to GC2. Less often, *comM* was interrupted by AbGRI1-type RIs carrying resistance genes exclusively within GC2 isolates (except M20), while AbaR4 was only found in M03 that belongs to the ST113^Pas^/2246^Oxf^. AbGRI1 was found in three variant structures shown in Figure 4. The first variant was AbaR4b, also called AbGRI1-1/*Tn2006* and Tn6166/*Tn2006*, that lacked the typical AbGRI1 structure [*Tn6022*-plasmid linker-*Tn6172*]. Instead, AbaR4b was formed of Tn*6166* in which Tn*6022*Δ*1* was interrupted by Tn*2006*. AbaR4b was identified in the chromosomes of GC2 isolates of the Oxford ST 1816/195. Another AbGRI1 variant, named here AbGRI1-like-1, was similar to AbGRI1-0, but Tn*6022* was replaced by Tn*6022*Δ*1*, and the integrase-coding gene (*int*) of the plasmid linker was uniquely interrupted by and IS*Aba125* element. The island carried three resistance genes (*sul2, strA*, and *strB*). Of all abGRI1-type RIs, AbGRI1-like-2 carried the largest number of resistance genes (*bla*_OXA−23_, *tet(B), sul2, strA*, and *strB*). Compared to AbGRI1-0, this version distinctively carried Tn*6022* in which *sup* gene was interrupted by Tn*2006*. In addition, [*tet(B)*-*tetR(B)*] element was also inserted within Tn*6172*.

Two types of IS*26*-bound genomic RIs were also identified. These included AbGRI2- and AbGRI3-type RIs that coexisted in all GC2 isolates. Only one version of AbGRI2 (AbGRI2-15) (Liepa et al., 2021) was identified. It harbored the two resistance genes *bla*_TEM−1_ and *aphA1*. While two versions of AbGRI3 were identified, an expanded form (designated before AbGRI3-1) was the dominant one (Blackwell et al., 2017). AbGRI3-1 carried seven resistance genes [*mphE-msrE-armA-sul1-aadA1-catB8-aacA4]*. A shorter version, designated before as AbGRI3-4 (Blackwell et al., 2017), was confined to GC2 isolates of the ST1816/195^Oxf^ with only three resistance genes onboard [*mphE, msrE*, and *armA*]. Bracketed by two inversely oriented copies of IS*Aba1*, GIsul2 RI carrying *sul2* as a sole resistance gene co-existed with Tn*6022* in all GC1 isolates and their phylogenetically related isolates M06 and M09.

As described above, RI-PER-7 was identified on a plasmid carried by M03 whose sequence was partially identified. The island hosted the largest combination of resistance genes compared to other RIs identified in the current study. These included *arr-2, cmlA5*, *bla*_PER−7_, *armA, msrE, mphE*, and two copies of *sul1*. RIs correlated to different STs are shown in Table 3.

**Table 3:** Correlation between genomic resistance islands and STs.

**GC**	**CC^**Pas**^**	**CC^**Oxf**^**	**ST^**Pas**^**	**ST^**Oxf**^**	**Isolate No**.	**RI**	**Resistance genes**	**References**
1	1	231	19	1,604/231	M12	Tn*6022*	None	Hamidian and Hall, 2011
					M15	GIsul2	*sul2*	Nigro and Hall, 2011
2	2	208	2	1,050/2,058	M01	AbGRI1-like-1	*sul2-strA-strB*	Hamidian and Hall, 2017b
				1,050/2,058	M16	AbGRI2-15	*bla*_TEM−1_ *-aphA1*	Liepa et al., 2021
				1,050/2,058	M17	AbGRI3-1	*mphE-msrE-armA-sul1-aadA1-catB8-aacA4*	Blackwell et al., 2017
				1,816/195	M04	AbaR4b	*strA*−*strB*−*tet*(*B*)−*bla*_OXA−23_	Seputiene et al., 2012
				1,816/195	M05	AbGRI2-15	*bla*_TEM−1_ *-aphA1*	Liepa et al., 2021
				1,816/195	M13	AbGRI3-4	*mphE-msrE-armA*	Blackwell et al., 2017
		546	570	1,701	M20	Tn*6022*	None	Hamidian and Hall, 2011
						AbGRI2-15	*bla*_TEM−1_ *-aphA1*	Liepa et al., 2021
						AbGRI3-1	*mphE-msrE-armA-sul1-aadA1-catB8-aacA4*	Blackwell et al., 2017
9	464	1,078	85	1,089	M02	Tn*6022*	None	Hamidian and Hall, 2011
					M11			
				1,078	M18			
-^a^	113	2,329	113	2,246	M03	AbaR4	*bla*_OXA−23_	Hamidian and Hall, 2011
						RI-PER-7	*arr-2-cmlA5-sul1* (2 copies) −*bla*_PER−7_*-armA-msrE-mphE*	Adams et al., 2020
				2,329	M14	AbGRI1-like-2	*strA*−*strB*−*tet*(*B*)−*sul*2−*bla*_OXA−23_	Bi et al., 2020
	164	234	164	1,418	M19	-	-	-
ND^b^	ND	ND	ND	ND	M06^c^; M09^c^	Tn*6022*	None	Hamidian and Hall, 2011
						GIsul2	*sul2*	Nigro and Hall, 2011
					M10^d^	-	-	-

*GC, international clone; CC, clonal complex; ST, sequence type; superscript Pas, Pasteur scheme; superscript Oxf, Oxford scheme; RI, resistance island*;

*^a^not belonging to any GC*;

*^b^could not be determined*;

*^c^phylogenetically related to GC1 isolates*;

*^d^phylogenetically related to M19 (ST164^Pas^/ST1418^Oxf^)*.

## Discussion

Updates on the genetic background of MDR and XDR *A. baumannii* are continuously being published from different parts of the world (Hamidian and Nigro, 2019; Gheorghe et al., 2021; Wareth et al., 2021) and from Egypt as well (Hassan et al., 2021; Jalal et al., 2021; Wasfi et al., 2021). However, reports about the association of resistance genes with RIs are relatively scarce. This is in part due to the need for multistep PCR mapping or whole-genome sequencing. To the best of our knowledge, this is the first report about the diversity and the genetic configuration of genomic RIs carried by *A. baumannii* isolates from Egypt. For this purpose, draft genomes of 18 non-duplicate MDR and XDR isolates were generated. Mostly from ICUs, the isolates were recovered from patients with bloodstream, respiratory tract, and wound infections. Very few treatment options were available. Draft genomes were employed for MLST analysis using both Pasteur and Oxford schemes. Oxford scheme-based analysis revealed the co-existence of two different alleles of the *gdhB* locus in 7/18 isolates generating two STs per isolate, a previously reported drawback for the scheme (Tomaschek et al., 2016). Nevertheless, MLST profiles generated by the Oxford scheme showed superior discrimination and concordance with the SNP-based phylogeny results. This was in line with other reports as well (Tomaschek et al., 2016; Gaiarsa et al., 2019). As reported before (Karah et al., 2012), most of the MDR and XDR *A. baumannii* strains belong to the Pasteur CCs 1 and two widely known as GC1 and GC2. In agreement with other studies (Al-Hassan et al., 2019; Fam et al., 2020; Wasfi et al., 2021), MLST analysis revealed the predominance of GC2 isolates in our collection (38.8%). Nevertheless, GC1 strains outweighed those that belonged to GC2 studied by others in our region (Ghaith et al., 2017; Jalal et al., 2021). Less representation (16.6%) was noted for the recently described GC9 (Müller et al., 2019) known to be endemic in Middle East countries (Al-Hassan et al., 2013, 2021; Bonnin et al., 2013; Ghaith et al., 2017; Jaidane et al., 2018; Salloum et al., 2018). Notably, a low prevalence of high-risk GCs was reported by older studies in Egypt (Al-Hassan et al., 2013; El Bannah et al., 2018), reflecting the progressive expansion of high-risk global clones in Egyptian hospitals over years. The emergence of successful STs not assigned to any high-risk GCs was also evident in the current study. These included the novel clonal complex CC2329^Oxf^ (CC113^Pas^) represented by M03 (ST113${}_{/}^{\text{Pas}}$2246^Oxf^) and M14 (ST113${}_{/}^{\text{Pas}}$ ST2329^Oxf^). Isolates that belong to this CC were reported from Egypt (Jalal et al., 2021), Saudi Arabia (Lopes et al., 2015), and Brazil (Leal et al., 2020). Another ST identified here, ST164^Pas^/1418${}_{,}^{\text{Oxf}}$ was reported in the isolates from Myanmar (Tada et al., 2020), Vietnam (Wareth et al., 2021), Brazil (Coelho-Souza et al., 2013), Kenya (Musila et al., 2021), and as a major clone in Thailand (Khuntayaporn et al., 2021). SNP-based phylogenetic analysis showed that our isolates were clustered with others from different parts of the world. Transmission of genetically related strains over continents might be facilitated by travel-associated fecal colonization (Ostholm-Balkhed et al., 2013) and medical tourism (Benenson et al., 2018). Together with international strains with similar STs, our isolates were distributed over seven clusters in a pattern that fully matched the Oxford scheme-based STs.

The detailed profiling of the resistance determinants and correlation to STs and resistance phenotypes was presented in the current study with special emphasis on resistance to β-lactams and aminoglycosides. The epidemiological linkage between specific *bla*_OXA−51−like_ variants and certain GCs was reported before (Zander et al., 2012; Karah et al., 2016; Jaidane et al., 2018) and was also evident in our isolates. This was noted for *bla*_OXA−64_,*bla*_OXA−66_,and *bla*_OXA−94_ that were linked to GC1, GC2, and GC9, respectively. In agreement with others (Jalal et al., 2021), *bla*_ADC−79_ was linked to GC1. Except for those that belonged to ST1050/2058^Oxf^ which carried *bla*_ADC−30_, GC2 isolates carried *bla*_ADC−73_, a single-nucleotide variant of *bla*_ADC−30_ (Karah et al., 2016). IS*Aba1-*amplified ADC-type β-lactamases, previously coupled to high-level cephalosporin resistance (Corvec et al., 2003), were confined to GC1 and GC2 isolates. Carbapenem resistance in our collection was mediated by two carbapenem-hydrolyzing enzymes, namely, OXA-23 and NDM-1. OXA-23 was the most common of all acquired β-lactamases produced by our isolates. It is also the most frequently described carbapenemase globally (Hamidian and Nigro, 2019). The gene *bla*_OXA−23_ existed in Tn*2006* that, in turn, was sometimes embedded in RIs. Despite the variety of the genetic platforms known to harbor *bla*_OXA−23_, Tn*2006* is the dominant vehicle for the acquisition of the gene worldwide (Nigro and Hall, 2016; Hamidian and Nigro, 2019). Less frequently, carbapenem resistance was mediated by NDM-1 (class B β-lactamases). In addition to one GC2 isolate (M20) and the phylogenetically related isolates M10 and M19, *bla*_NDM−1_ was carried by all GC9 (ST85^Pas^) isolates. GC9 was claimed to act as a reservoir for *bla*_NDM−1_ in our region (Bonnin et al., 2013; Jaidane et al., 2018; Salloum et al., 2018; Al-Hassan et al., 2021). As described in our previous study (Zafer et al., 2021), *bla*_NDM−1_ laid within an IS*Aba14*-bracketed transposon also carrying *aphA6* in M02, M11, and M10. However, the transposon could not be identified in other NDM-producer strains in our collection. Large-scale screening of this novel transposon is, therefore, recommended. Although reported in a higher prevalence in Egypt (El-Sayed-Ahmed et al., 2015; Abouelfetouh et al., 2019; Wasfi et al., 2021), a combination of the carbapenemase-coding genes *bla*_OXA−23_ and *bla*_NDM−1_ was found in only one isolate (M20). Class A β-lactamases, including TEM-1, CARB-16, and PER-7, had a considerable share in our collection too. While the exact environment of *bla*_CARB−16_ could not be identified, both *bla*_TEM−1_ and *bla*_PER−7_ were exclusively carried within RIs, including AbGRI2 and RI-PER-7, respectively. The latter was first described as Tn*1548*-like-2 transposon by Karah et al. (2016) and later designated RI-PER-7 by Adams et al. (2020). Both reported the island in isolates that had Pasteur ST25, a double-locus variant of ST113^Pas^ in which RI-PER-7 was identified here. Shorter versions of the island were also reported to be carried by members of the family *Enterobacteriaceae* (Adams et al., 2020). WGSs of four isolates of the ST ST2246^Oxf^ were generated in a recent study in Egypt (Jalal et al., 2021). While the authors did not report the RI-PER-7 in their isolates, the signature genes of the island [*mphE, msrE, armA, sul1*, *bla*_PER−7_, *cmlA5*, and *arr2*] were identified in 3/4 (75%) isolates. This newly emerging ST might thus serve as a reservoir for RI-PER-7. While the RI-PER-7-positive contig of M03 showed the highest similarity to a plasmid sequence and was thus anticipated to be plasmid-mediated, the exact location of the island in ST2246^Oxf^ is yet to be confirmed.

Of all the aminoglycoside resistance genes identified here, those conferring resistance to the clinically relevant aminoglycosides, amikacin, gentamicin, and tobramycin, were of much concern. Most importantly, the broad-spectrum *armA* gene conferring high-level resistance to all aminoglycosides (Galimand et al., 2003) was carried by all GC2 isolates. A high prevalence of *armA* has been previously reported in Egypt (El-Sayed-Ahmed et al., 2015). In addition, co-existing *bla*_OXA−23_, *bla*_NDM−1_, and *armA* were found in one GC2 isolate (M20). This combination was identified on plasmids of 8/25 (32%) isolates in an older study in Egypt (El-Sayed-Ahmed et al., 2015). Amikacin resistance in our isolates was also coded by *aphA6* in 38.8% of the isolates. The gene is commonly identified in *A. baumannii* within the composite transposon Tn*aphA6* in which it is bracketed by two directly oriented IS*Aba125* elements (Nigro et al., 2011). Nevertheless, here it was most commonly identified within a bracket of an upstream IS*Aba14* element and a downstream IS*Aba125* element that was sometimes part of a composite transposon also carrying *bla*_NDM−1_, as described above. Given that the IS*Aba125* elements are thought to drive the overexpression of *aphA6* imparting high-level amikacin resistance, the effect of the upstream IS*Aba14* insertion is questionable. Notably, this unusual environment was associated with unrelated STs. While frequently found on plasmids (Nigro and Hall, 2012b; Hamidian and Hall, 2014; Hamidian et al., 2014), plasmid*-*associated *aphA6* was found in one isolate (M05) in our collection. Encoding resistance to both gentamicin and tobramycin, *aadB* was spotted in 7/18 isolates on pRAY plasmid derivatives. In *A. baumannii, aadB* was identified within a class 1 integron or more commonly on the globally distributed pRAY plasmid derivatives (Hamidian et al., 2012). While the GC1 isolates M12 and M15 did not carry any pRAY plasmids or *aadB*, such plasmids were carried by their phylogenetically related isolates M06 and M09. Other pRAY-positive STs included ST85^Pas^ and ST164^Pas^. In addition to *armA*, the long version of AbGRI3-1 RIs also carried *aac4* that encodes the aminoglycoside modifying enzyme AAC(6')-Ib', conferring resistance to gentamicin rather than amikacin modified by AAC(6')-Ib (Ramirez and Tolmasky, 2010). In agreement with a recent study from Egypt, *aac4* was merely carried by a subset of GC2 strains, perhaps carrying the same version of AbGRI3 (Jalal et al., 2021). In addition, a wide array of resistance genes to other antimicrobial classes including multidrug efflux pumps was abundantly identified in our collection.

The advances in the next-generation sequencing technology made possible the genome-wide resistome analysis of different bacterial species and the investigation of the association of resistance genes with mobile genetic elements. Despite the difficulties imparted by the fragmented nature of contigs assembled from the short-read sequencer output, we used a combined approach of *de novo* assembly and reference mapping to uncover the configurations of RIs carried by our isolates. Examining the integrity of the *comM* gene, the hotspot for insertion of AbaR, AbaR4, and AbGRI1-type islands was the first step in RI analysis. The gene was found to be interrupted in the majority of the isolates (16/18) equally by the resistance genes-free transposon Tn*6022* and either AbaR4 or AbGRI1-like RIs (44.4%). A similar prevalence (46.7%) was reported for Tn*6022* in *A. baumannii* isolates from Korea (Kim et al., 2017). Tn*6022*, the backbone of AbaR4, comprises five transposition genes (*tniCABDE*), a universal stress protein-encoding gene (*uspA*), and a sulfate permease-coding gene (*sup*) (Hamidian and Hall, 2011). It has been widely identified in lineage 2 GC1 isolates (Hamidian and Hall, 2011). Here, the transposon was carried by GC1 isolates and their phylogenetically related ones (M06 and M09). In addition, the transposon interrupted the *comM* gene in all GC9 isolates and only one GC2 isolate (M20). In Tn*6022*, the gene *sup* is a hotspot for insertion of Tn*2006* with an embedded *bla*_OXA−23_ generating AbaR4. While widely described in GC2 isolates (Kim et al., 2012, 2013), AbaR4 was identified here within the chromosome of M03 only. Interestingly, co-existing chromosomal and plasmid copies of AbaR4 were recently reported in *Proteus mirabilis* (Octavia et al., 2020). While not self-transmissible (Bi et al., 2019), AbaR4 mobility to the chromosome of *P. mirabilis* was proposed to be mediated by plasmids (Octavia et al., 2020). This demonstrates the impending threat of interspecies plasmid-mediated transfer of AbaR4.

Twenty-two AbGRI1 configurations with different backbones were characterized by Bi et al. (2020). Here, we identified three variant structures of AbGRI1. Of them, two variants had the typical AbGRI1 backbone [Tn*6022* (or the deletion derivative, Tn*6022*Δ)-linker-Tn*6172*]. Despite their unique configurations, they were named here as AbGRI1-like-1 and AbGRI1-like-2 due to the lack of a universal nomenclature system for *A. baumannii* genomic RIs (Hamidian and Hall, 2018). AbGRI1-like-1 was identified in ST1050/2058^Oxf^ GC2 isolates. It was made up of Tn*6022*Δ, a plasmid-derived linker with *int* gene uniquely interrupted by an IS*Aba125* element and Tn*6172*. The IS*Aba125* element interrupting the *int* gene might act as a hotspot for the insertion of resistance elements flanked by two direct copies of IS*Aba125* (such as Tn*aphA6*) by homologous recombination. The second variant AbGRI1-like-2 was formed of AbaR4, plasmid-derived linker, and Tn*6172* to which a ΔIS*CR2*-ΔTn*10* fragment carrying *tet(B)* gene was inserted (Hamidian and Hall, 2017b). This configuration resembled that of an unnamed RI identified by Bi et al. (2020) that also carried a second copy of AbaR4 inserted within *tet(B)* gene. AbGRI1-like-2 was solely carried by M14. The third variant of AbGRI1 was a Tn*2006*-interrupted AbGRI1-1. It had an atypical AbGRI1 structure lacking the plasmid-derived linker. Even though AbaR4 was reported not to have any variants (Hamidian and Hall, 2018), the Tn*2006*-interrupted AbGRI1-1 was named by Seputiene et al. (2012) as AbaR4b. For convenience, this name was used in the context of this study. In addition to conferring resistance to carbapenems, AbaR4b confers resistance to tetracycline and streptomycin/spectinomycin and was first identified in *A. baumannii* strains from Lithuania (Seputiene et al., 2012). In Korea, AbaR4b was found in 22.4% of the tested isolates, and all belonged to GC2 (Kim et al., 2017).

The IS*26*-bracketed RIs AbGRI2 and AbGRI3 co-existed in all GC2 isolates. While AbGRI2 occurred in only one configuration (AbGRI2-15), two variants of AbGRI3 with different resistance spectra were found. AbGRI2 has been reported most commonly in GC2 isolates carrying all or some of the resistance genes *bla*_TEM_*, aphA1, catA1*, and [*sul1, aadA1*, and *aacC1*] within a class I integron (Nigro et al., 2013). AbGRI2-15 was first identified by Liepa et al. (2021) in *A. baumannii* strain collected from a patient in Lebanon. The island is a short version of AbGRI2 in which only *aphA1* and *bla*_TEM−1_ remained after IS*26*-mediated deletion and recombination events, as reported by the author. Each of the GC2 isolates carried either of the two variants AbGRI3-1 or AbGRI3-4. AbGRI3-1, also known as Tn*6180*, was first identified in *A. baumannii* strains MDR-TJ (GenBank accession: CP003500.1) and TYTH-1 (GenBank accession: CP003856.1) conferring resistance to four classes of antimicrobial agents (Blackwell et al., 2017). It was the least frequently identified among AbGRI3-type islands identified in a collection of *A. baumannii* isolates from Singapore in a study by Blackwell et al. (2017). In the same study, AbGRI3-4 was the dominant one identified in 46.6% of isolates. AbGRI3-4 is a short version of AbGRI3, most importantly, retaining *armA*. Blackwell et al. (2017) also highlighted the successful dispersion of AbGRI3-4 in isolates from India and Sweden.

Genomic island sul2 (GIsul2) is an integrating element first described by Nigro and Hall (2011) in the chromosome of *A. baumannii* strain ATCC 17978 isolated in France in 1951. Within GIsul2 is located a dihydropteroate synthase type 2-coding gene (*sul2*) preceded by an IS*Aba1*. While thought to provide a promotor for *sul2* (Nigro and Hall, 2011), high-level sulfonamide resistance in ATCC 19606 conferred by *sul2* was not accompanied by an upstream IS*Aba1* (Hamidian and Hall, 2017a). GIsul2 was reported as the main vehicle for the mobilization of the *sul2* gene (Hamidian and Hall, 2017a). It is also the donor of [CR2-*sul2*] elements in AbGRI1-type RIs integrated through homologous recombination. Here, GIsul2 was solely identified in GC1 isolates and those found to be phylogenetically related.

## Conclusion

Beyond merely defining the resistance genes standing behind MDR and XDR in *A. baumannii*, WGS was used in the current study for defining the genomic RIs carried by clinical isolates of *A. baumannii* from Egypt. Epidemiology analysis of the isolates showed a significant representation of the high-risk GCs, particularly GC2. Novel ST (ST2329^Oxf^), resistance gene (*bla*_ADC−258_), and IS (IS*Aba61*) were identified. RIs were carried by the majority of the isolates most frequently co-existing in GC2. They were loaded with several genes conferring resistance to various antimicrobial classes and hotspots for the acquisition of more resistance genes. AbGRI1-type RIs showed up with the widest diversity, including two novel configurations.

## Data Availability Statement

The datasets presented in this study can be found in online repositories. The names of the repository/repositories and accession number(s) can be found in the article/Supplementary Material.

## Ethics Statement

The study was performed in accordance with relevant guidelines and regulations, and no experiments were performed on humans and/or human tissue samples. The study was approved by the local Ethical Committee of the Clinical and Chemical Pathology Department, Kasr Al-Ainy Hospital, Cairo University. Bacterial isolates were only collected as part of the routine patient care, and informed consent was not required.

## Author Contributions

MZ, AH, MA-A, HR, and SH contributed to the study design, performance of experiments, and data analysis. SH performed the genome assembly, bioinformatics analysis, and wrote the first draft of the manuscript. All authors read and approved the final version of the manuscript.

## Funding

The authors thank the Deanship of Scientific Research at King Saud University for funding this work through Project No. RGP-038.

## Conflict of Interest

The authors declare that the research was conducted in the absence of any commercial or financial relationships that could be construed as a potential conflict of interest.

## Publisher's Note

All claims expressed in this article are solely those of the authors and do not necessarily represent those of their affiliated organizations, or those of the publisher, the editors and the reviewers. Any product that may be evaluated in this article, or claim that may be made by its manufacturer, is not guaranteed or endorsed by the publisher.
