# Supplementary material for: Genetic Configuration of Genomic Resistance Islands in Acinetobacter baumannii Clinical Isolates From Egypt
Source: Front Microbiol. 2022 Jul 22;13:878912. doi: 10.3389/fmicb.2022.878912 (PMC9353178; doi:10.3389/fmicb.2022.878912)
Supplement: Supplementary file 1 [file Table_1.DOCX]

Supplementary Material

**Supplementary Table 1: Post assembly and annotation metrics of *Acinetobacter baumannii* strains sequenced in the current study**

| Isolate  Number | Post assembly metrics | | | | | Annotation metrics | | | | |
| --- | --- | --- | --- | --- | --- | --- | --- | --- | --- | --- |
|  | # contigs | Total length | GC (%) | N50 | Genome coverage | Total genes | Total CDSs | Coding genes | CDSs with proteins | RNA genes |
| M01 | 427 | 3898839 | 39.19 | 17854 | 19X | 3,853 | 3,780 | 3,690 | 3,690 | 73 |
| M02 | 455 | 3919334 | 39.19 | 16577 | 16X | 4,068 | 3,996 | 3,878 | 3,878 | 72 |
| M03 | 691 | 4168367 | 39.38 | 11611 | 18X | 4,250 | 4,188 | 4,030 | 4,030 | 62 |
| M04 | 319 | 3860276 | 39.1 | 30752 | 19X | 3,792 | 3,720 | 3,650 | 3,650 | 72 |
| M05 | 807 | 3846906 | 39.43 | 8540 | 18X | 3,939 | 3,880 | 3,775 | 3,775 | 59 |
| M06 | 1375 | 3516560 | 39.79 | 3737 | 20X | 4,419 | 4,368 | 4,020 | 4,020 | 51 |
| M09 | 1226 | 3564520 | 39.83 | 4487 | 26X | 4,544 | 4,492 | 4,165 | 4,165 | 52 |
| M10 | 1967 | 2906735 | 40.52 | 1894 | 32X | 4,450 | 4,418 | 3,992 | 3,992 | 32 |
| M11 | 690 | 3834431 | 39.49 | 10028 | 24X | 3,823 | 3,761 | 3,628 | 3,628 | 62 |
| M12 | 416 | 3831373 | 39.25 | 19228 | 28X | 3,792 | 3,723 | 3,621 | 3,621 | 69 |
| M13 | 521 | 3895139 | 39.12 | 15153 | 16X | 3,895 | 3,832 | 3,736 | 3,736 | 63 |
| M14 | 774 | 4085551 | 39.47 | 9127 | 18X | 4,219 | 4,153 | 3,988 | 3,988 | 66 |
| M15 | 306 | 3875831 | 39.17 | 29752 | 49X | 3,870 | 3,799 | 3,693 | 3,693 | 71 |
| M16 | 294 | 3940454 | 39.1 | 27088 | 25X | 3,863 | 3,790 | 3,712 | 3,712 | 73 |
| M17 | 181 | 3967811 | 38.99 | 41881 | 32X | 3,877 | 3,804 | 3,736 | 3,736 | 73 |
| M18 | 396 | 4006963 | 39.07 | 26535 | 63X | 4,057 | 3,984 | 3,876 | 3,876 | 73 |
| M19 | 908 | 3773846 | 39.55 | 7080 | 42X | 3,870 | 3,808 | 3,642 | 3,642 | 62 |
| M20 | 481 | 3899209 | 39.41 | 15553 | 42X | 3,873 | 3,796 | 3,700 | 3,700 | 77 |


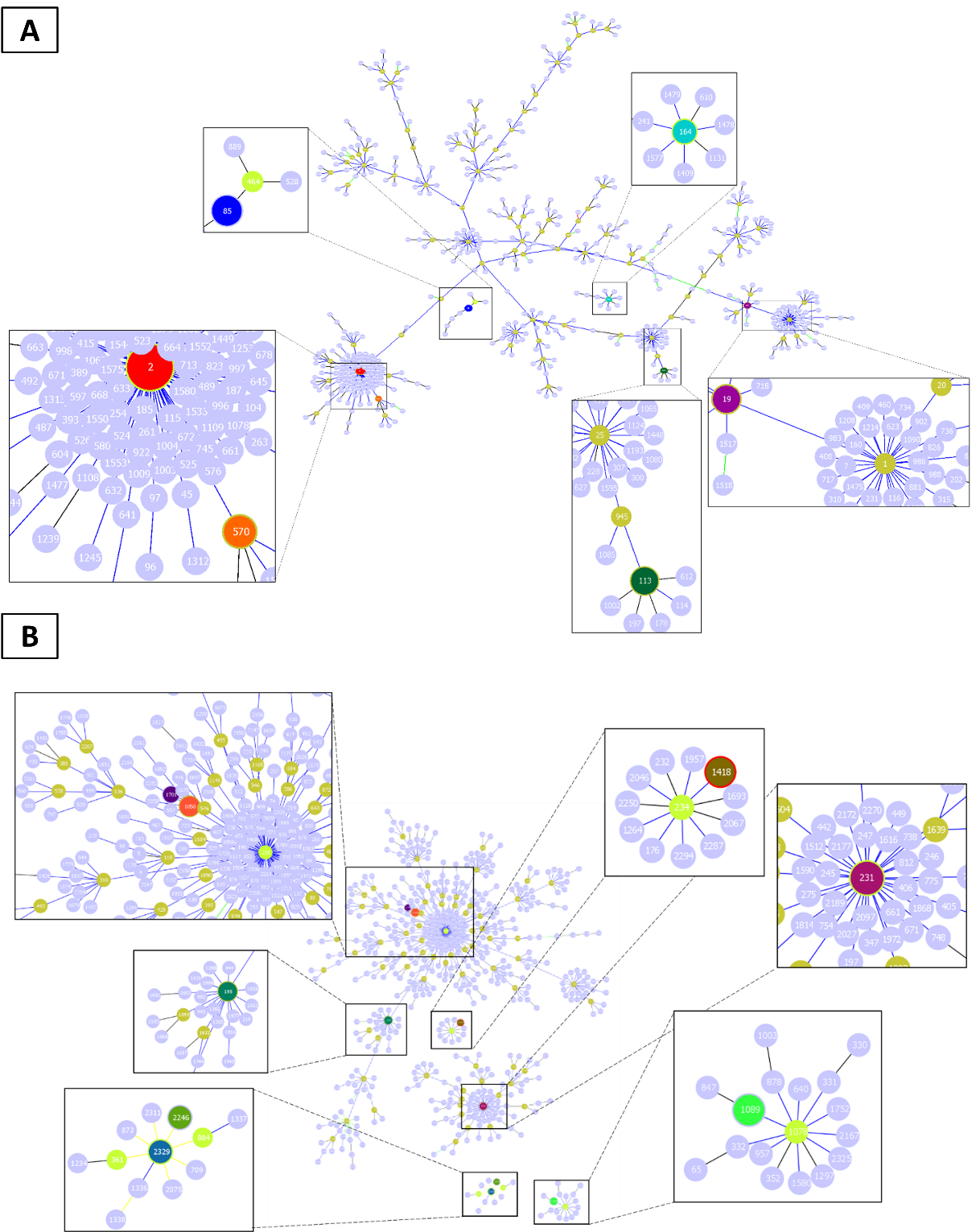


Supplementary Figure 1. Minimum Spanning Tree diagram of the STs of *A. baumannii* strains sequenced in the current study together with other STs in MLST database. (A) STs of Pasteur scheme. (B) STs of Oxford scheme. Numbers inside the circles refer to STs. Light blue and light green circles correspond to STs in the database and clonal complexes, respectively. Circles of other colors correspond to the STs to which the strains sequenced here belong.


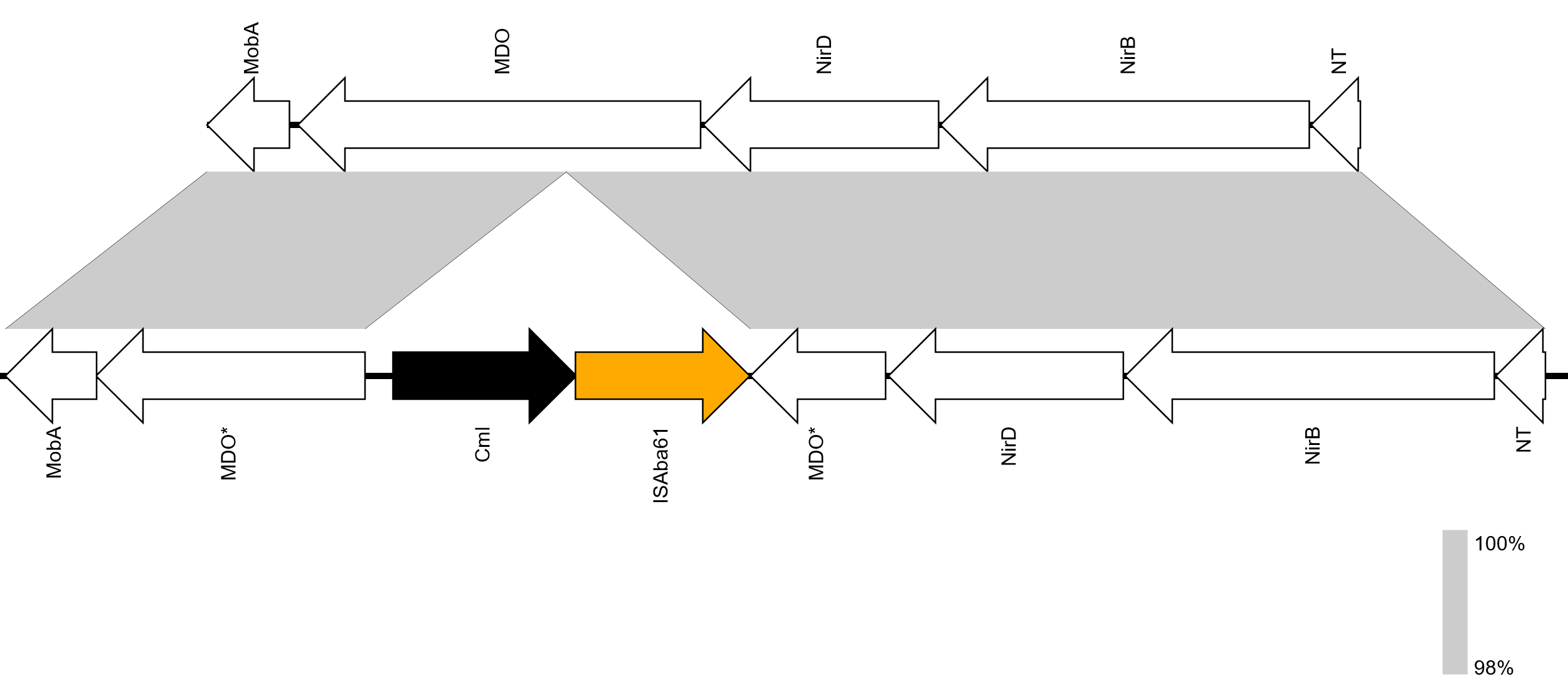


**Supplementary Figure 2**. **Depiction of the insertion site of the novel IS*Aba61* and the passenger *cmlA*/*floR*-like gene.** ORFs orientation is indicated by arrows. Grey bands between panels indicate more than 98 % sequence similarity. Genes are labelled by their protein products;

NT, Nitrate/Nitrite transporter; NirD, nitrite reductase small subunit; NirB, nitrite reductase large subunit; MDO*, molybdopterin-dependent oxidoreductase (interrupted); Cml, CmlA/FloR family chloramphenicol efflux MFS transporter; ISAba61, ISL3 family transposase; MobA, Molybdopterin-guanine dinucleotide biosynthesis protein
